# Health sciences librarians supporting health and nutrition education in a culinary medicine curriculum

**DOI:** 10.5195/jmla.2020.911

**Published:** 2020-10-01

**Authors:** Trey Lemley, Rachel Finch Fenske

**Affiliations:** 1 lemley@southalabama.edu, Senior Librarian, Information Services Librarian, and Head of Reference and Collection Development, Charles M. Baugh Biomedical Library, University of South Alabama, Mobile, AL; 2 rfenske@southalabama.edu, Assistant Librarian and Information Services/Outreach Librarian, Charles M. Baugh Biomedical Library, University of South Alabama, Mobile, AL

## Abstract

**Background:**

Culinary medicine is an innovative approach to teaching health sciences students and other health professionals the basics of healthy eating, food preparation, and nutrition through applied instruction. It is hoped these professionals will, in turn, share their knowledge with patients. The University of South Alabama Mitchell Cancer Institute licensed the Tulane University's Goldring Center for Culinary Medicine curriculum and began teaching medical, nursing, and other health sciences students as well as community members in 2017. The authors describe a collaboration between librarians and health professionals to connect with underserved community members by teaching the basics of good nutrition and healthy meal preparation.

**Case Presentation:**

Two health sciences librarians provided instruction to community members in the use of quality health information resources during various modules of the culinary medicine curriculum. Demonstrations of the use of MedlinePlus and ChooseMyPlate were conducted using topics from module content. Evaluations were distributed after each module to evaluate the effectiveness of the library component, the results of which enabled librarians to subsequently increase their instruction time and implement iPad use for more engaging participation.

**Conclusion:**

Librarians were seen as invaluable partners in this innovative program and became an integral part of the curriculum. Evaluation results helped librarians advocate for more instructional time. As a result of their involvement, librarians were given additional outreach opportunities to educate younger populations at risk of developing chronic health diseases.

## BACKGROUND

Culinary medicine is a new approach to health education that is becoming popular in the curricula of medical, nursing, and other health sciences schools. Its goal is to help health care providers empower their patients with the knowledge to combat and prevent chronic illnesses so that patients can make good decisions about their eating habits, food consumption, food selection, and food preparation. As defined by La Puma, culinary medicine is “a new evidence-based field in medicine that blends the art of food and cooking with the help of medicine” [1]. Dieticians and other health care providers provide information on specific diet plans and give sound advice for patients; however, many providers do not have the training or understanding of how healthy meal selection and preparation can assist in the overall well-being of a patient.

Culinary medicine helps both patients and health care providers understand the mechanics of food and how the choice of food can help improve various health conditions. When patients are told to change their diets, they are not generally given explanations in detail of why certain foods are better than others from their physicians. Culinary medicine attempts to provide an understanding not only of eating behaviors, but also how food “influences metabolism, immunity, pathophysiology and well-being” [1]. Empowering patients to take a more informed approach to what they eat and how they prepare food provides them with a better understanding of the role food plays in everyday living. When healthy food consumption can be seen as a treatment method for reducing elements of disease such as high cholesterol, patients will begin to associate food as medicine [1].

Tulane University's Goldring Center for Culinary Medicine is the pioneer in the field of culinary medicine. The first of its kind, the Goldring Center is a medical school–based teaching initiative led by a physician and trained chef that addresses deficiencies in nutrition education competencies among newly trained physicians [2]. The Goldring Center has developed a culinary medicine curriculum to teach nutrition and cooking skills to medical students. Currently, more than fifty academic medical centers including medical schools, nursing schools, and residency programs use the Goldring Center's curriculum. To illustrate, medical students at the University of Central Florida who participate in the Goldring Center curriculum not only learn culinary skills, but also transfer this knowledge to provide practical solutions for their future patients. Presented with patient case scenarios, medical students are “asked to determine how food choices ‘can impact and help heal'” [3]. In addition, the Goldring Center convenes yearly conferences to expand knowledge in this evolving curriculum, showcasing cooking skills, research forums, and panel discussions on the importance of healthy eating and good health [4].

The primary goal of the curriculum is to educate participants in the preparation of nutritious, tasty, and affordable food. Using the Mediterranean diet as its basis, the curriculum includes topics on nutrition and health, kitchen sanitation and workflow, healthy food shopping, weight management, and metabolic risk factors [4]. Participants learn portion control, how to read food labels, and culinary behaviors, such as preparing and cooking breakfast or using beans and legumes as part of a nutritious diet [4, 5]. According to Birkhead et al., participants benefit from practical instruction in the kitchen or classroom on changing eating habits, thus setting the stage for collaborative patient/physician relationships as participants engage in healthy eating and enjoy good health [2]. Talking to patients about the practicality of preparing and choosing good food can help reduce chronic disease and create conversations in the exam room that help patients understand the importance of healthy eating and nutrition. Since 2010, the curriculum has been offered to over 6,000 community participants in New Orleans.

## CASE PRESENTATION

The University of South Alabama (USA) Mitchell Cancer Institute (MCI) licensed the culinary medicine curriculum from Tulane University's Goldring Center in 2016. To fund the program, MCI obtained a Well-Integrated Screening and Evaluation for WOMen across the Nation (WISEWOMAN) grant from the Centers for Disease Control and Prevention via the Alabama Department of Public Health. The WISEWOMAN program focuses on low income, uninsured, or underinsured women aged forty to sixty-five years [6]. Classes were taught collaboratively onsite at Bishop State Community College (BSCC) in Mobile, Alabama.

MCI is an academic cancer treatment center and research enterprise. Since its founding, MCI has become an integral part of the health care community and a much-needed resource for the central Gulf Coast region, performing interdisciplinary cancer treatment, a wide variety of clinical trials, and both basic and translational research. In addition, MCI makes community outreach a major priority given the health disparities that exist in the surrounding community. Because of its mission, MCI actively promotes activities aimed at cancer control and prevention.

After licensing the Goldring Center curriculum, MCI disseminated information about the program to potential stakeholders at USA and in the Mobile area who, in turn, immediately saw its value and possible benefit to medically underserved women in the community. Although the initiative at Tulane was aimed at medical students, numerous groups in Mobile expressed interest in collaborating, and the culinary medicine program expanded to be a joint effort among several stakeholders, including USA, BSCC, and members of the Mobile area community. Under the auspices of USA, a program administrator and a registered dietitian from MCI, two librarians from the Charles M. Baugh Biomedical Library, and faculty and students from the Colleges of Allied Health, Medicine, and Nursing participated.

Librarians' involvement included providing instruction on locating current, reliable, and high-quality information on nutrition and other components of a healthy lifestyle to help participants gain a better understanding of their health and dietary needs and enable them to make beneficial lifestyle changes. Faculty members from the Colleges of Allied Health, Medicine, and Nursing enlisted a small cohort of students to become immersed in the curriculum so that they could gain nutrition competencies while learning how to connect with their patients in a more effective way. Learning how to do this by cooking was a novel way of integrating nutritional knowledge with everyday applications for meal planning and execution.

BSCC was the site location where classes were taught, because it had an established Commercial Food Service Program and a teaching kitchen that accommodated sixteen people. The director of the Commercial Food Service Program was a trained chef and graduate of the College of Culinary Arts at Johnson and Wales University. She and other Commercial Food Service Program faculty assumed key leadership roles and participated in every class.

Since the culinary medicine program was initiated, there have been numerous cohorts of participants, who have consisted of community members or USA health sciences students, depending on the decisions of program leaders. During the first year of our involvement, participants consisted of community members from the Mobile area. In particular, Franklin Primary Health Center in Mobile encouraged their patients to participate in the program. Incentives to enroll, such as health progress monitoring over the course of a year and a one-time yearly gym membership, were provided.

### Curriculum

**Figure 1:**
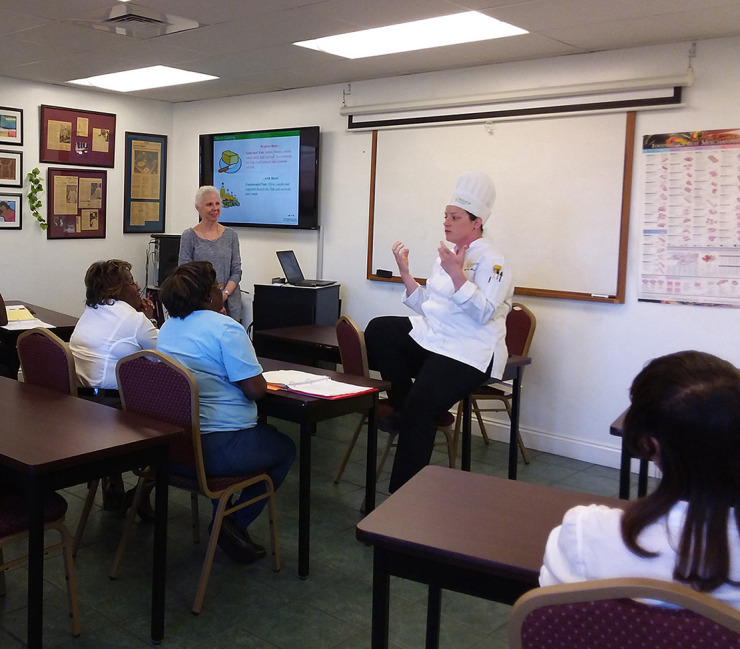
Chef providing classroom instruction for meal preparation

**Figure 2:**
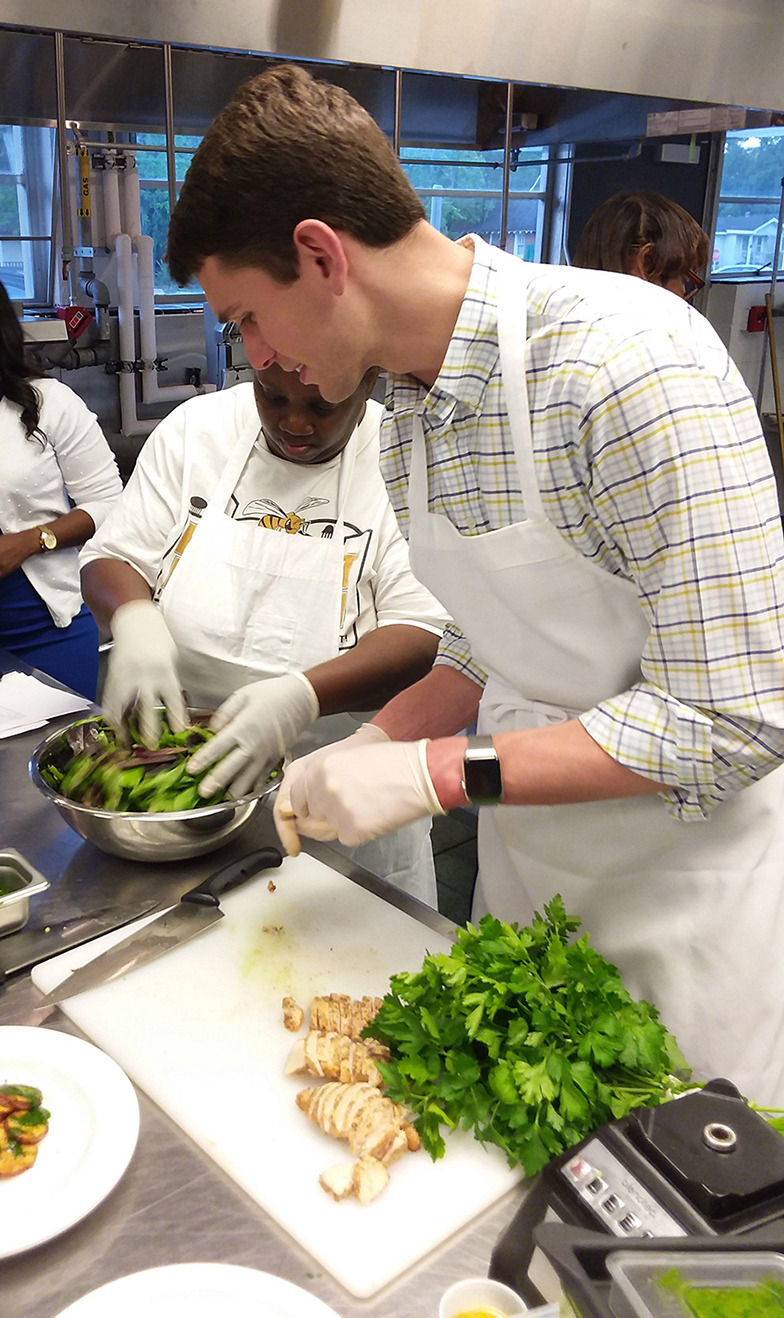
University of South Alabama (USA) medical student helping with food preparation

**Figure 3:**
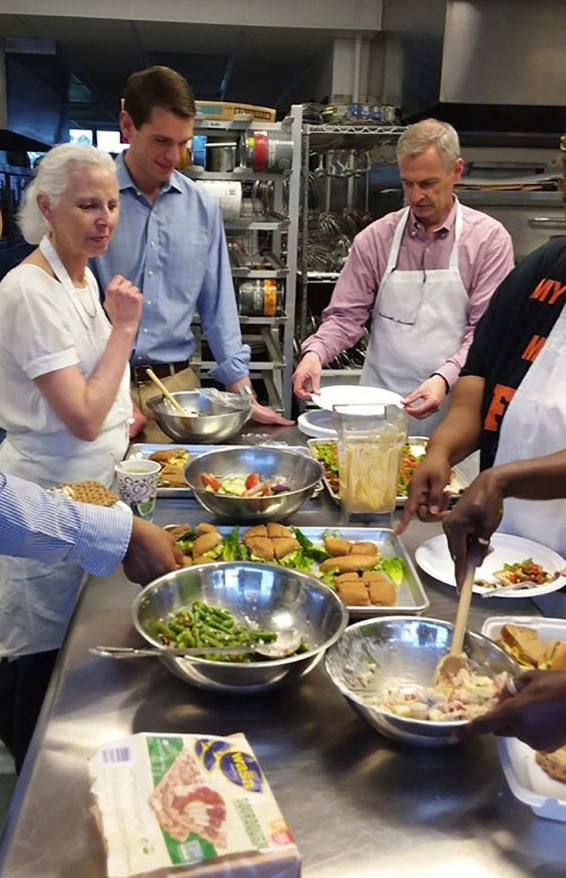
Culinary medicine instructors, librarian, and participants share meal

The culinary medicine curriculum comprised 6 modules per cohort, with classes held once a week from 5:30 p.m.–7:30 p.m. over a 6-week period. Each module commenced with a discussion led by culinary medicine faculty about the instructional content covered in the module (Figure 1), followed by actual meal preparation (Figure 2). All attendees then shared the meal, during which time the chef discussed the meal's nutritional content (Figure 3). The module concluded with an interactive discussion on how the meal preparation could be incorporated into the participants' weekly meal rotation.

The six modules include:

1. Introduction to Healthy Eating: Mediterranean Diet
2. Healthy Breakfasts and Nutrition Labels
3. Introduction to Healthy Eating
4. Healthy Lunches
5. Challenges to Healthy Eating: Carbohydrates and Healthy Snacking
6. Healthy Eating Resources and Soups and Salads

### Librarian involvement

One of the authors (Lemley), a senior librarian, was a long-time member of MCI's Cancer Control & Prevention Committee. In 2015, committee members were invited to attend an introductory meeting to learn about the culinary medicine program, during which time it was decided to license the curriculum from the Goldring Center. In 2016, the librarian asked MCI program officers if they would allow librarians to participate by providing consumer health information to participants in the curriculum. MCI enthusiastically embraced this idea and was very supportive. During that year, the librarian attended several planning sessions with major stakeholders. When onsite involvement began in 2017, the lead author (Lemley) invited the second author (Fenske), the new outreach services librarian, to join the initiative as a way to expand her contacts in the community. This involvement proved to be very successful, as additional outreach activities were initiated by virtue of participation in this project.

In all cohorts, library instruction sessions were based on teaching objectives for a particular module and were tailored to address the concepts introduced in the module. For example, when a module included materials on the Mediterranean diet, we demonstrated how to find information on this diet using the Health Topics category in MedlinePlus, a consumer-friendly health database from the National Library of Medicine. In response to questions from participants, we demonstrated how to access the MedlinePlus medical encyclopedia to explain unfamiliar terminology. Because module 4 included materials on dietary fiber, we demonstrated how to easily obtain quality information on this topic by reading the “Dietary Fiber” entry in the MedlinePlus Health Topics section. Finally, we introduced participants to ChooseMyPlate, an online resource from the US Department of Agriculture. At the end of our presentation, we distributed informational packets with pamphlets and brochures about MedlinePlus and other relevant health resources for participants' use.

Throughout the presentation, we emphasized that MedlinePlus and ChooseMyPlate were free, easy to use, current, and of the highest quality, and are produced by major federal agencies. During the first year of our involvement, the time frame of the library session was very brief, no more than fifteen minutes. This was an extremely short time period to demonstrate the resources; however, we were able to introduce the resources and make participants aware of their ease of use and quality. With this limited time period, we did not have the opportunity to engage in hands-on activities to reinforce concepts that were introduced in the module. However, we appreciated the opportunity to participate in the program and, by doing so, were hopeful that participants would use these resources to find current and reliable consumer health information relevant to good nutrition and a healthy lifestyle.

### Assessment

In 2017, the first year of our involvement, we participated in 2 cohorts (n=16), both consisting entirely of community members. After each presentation, we distributed a short evaluation survey (supplemental appendix) to participants. The purposes of this evaluation were to determine the possible value and benefit of the library component of the program and to gauge whether participants planned to apply the information to their specific health needs after the program. In addition, we wanted to justify the need for additional class time to integrate hands-on activities.

Our evaluation results suggested that most participants felt that the library component of the program was beneficial and that the information presented was new to them (Table 1). Most participants agreed that MedlinePlus was a valuable, easy-to-use, consumer-friendly health resource that would empower them to apply the information for their specific health needs. Although our instructional sessions were short (i.e., fifteen minutes), we were pleased to see that participants indicated that they would begin using the presented resources for their health information needs and would recommend the websites to their friends and family, thus promoting the use of quality health resources by a greater population.

**Table 1:** Library instruction evaluation results, 2017 cohorts

	Strongly agree	Somewhat agree	Somewhat disagree	Strongly disagree	Not applicable
The session led by the librarians introduced me to 1 or more health information sources or tools that I had never used before.	62.50%	37.50%	—	—	—
I think I can apply the information I learned in the MedlinePlus introduction session to my health and nutritional needs.	87.50%	12.50%	—	—	—
The information presented was helpful.	75.00%	25.00%	—	—	—
The information presented was clear and well organized.	75.00%	25.00%	—	—	—
I plan to start using MedlinePlus to locate health and nutrition information.	68.75%	31.25%	—	—	—
I would recommend MedlinePlus to my friends and family.	68.75%	31.25%	—	—	—
The amount of time devoted to the MedlinePlus session was adequate.	62.50%	31.25%	—	—	—
I would like to have more time learning how to use MedlinePlus.	56.25%	31.25%	6.25%	—	6.25%
I would like to have a hands-on session using MedlinePlus in the Culinary Medicine curriculum.	68.25%	18.75%	6.25%	—	6.25%
Having the library component in the Mitchell Cancer Institute (MCI) Culinary Medicine curriculum was beneficial.	62.50%	37.50%	—	—	—

Another beneficial discovery was that participants wanted more time to explore the health resources and indicated that a hands-on session would be helpful. Although participants indicated the class time allotted for teaching was adequate, responses to two additional questions gauging participants' interest in having more time to learn the databases and participate in a hands-on activity provided evidence that our allotted time was not sufficient. With these data, we were able to justify more instruction time in future cohorts to program administrators.

Informal comments from program officials, faculty, and participants regarding our lectures and participation in numerous cohorts from 2017 to 2019 have been very positive. Participants were excited to see librarians included in the curriculum and were given a new perspective on the work that health sciences librarians performed. Based on the 2017 evaluation results and positive reinforcement from program officials, we participated in additional cohorts in 2018 and 2019 and were asked to continue our involvement with three additional cohorts in 2020, all of which were cancelled due to the COVID-19 pandemic. Beginning with the 2019 cohort, library instruction sessions expanded to a thirty-minute segment and included a hands-on component using iPads.

Since implementing these changes in 2019, we distributed another evaluation form and found that participants, consisting of both community members and health sciences students (n=14), liked having a hands-on component, and many wanted more time to learn about MedlinePlus (Table 2). Therefore, the use of evaluation tools to assess the library sessions was beneficial for justifying our changes to the curriculum.

**Table 2:** Evaluation of hands-on activities, 2019 cohort

	Strongly agree	Somewhat agree	Somewhat disagree	Strongly disagree	Not applicable
I liked having the hands-on session using iPads to search MedlinePlus.	78.60%	14.30%	7.10%	—	—
I would like to have more time learning how to use MedlinePlus.	64.30%	28.60%	7.10%	—	—

### Outreach impact

Because of our positive impact in the curriculum, other avenues of instruction and outreach surfaced for the outreach librarian. She was invited to teach instructional sessions on MedlinePlus and other National Institutes of Health resources to undergraduate nutrition students at BSCC and taught a high school dietetics class about health issues and concerns that were prevalent in the community.

## DISCUSSION

The adoption of the Culinary Medicine program at USA's MCI created a unique opportunity for health sciences librarians to become full partners in this multidisciplinary approach to healthy eating. We were seen as valuable allies in curriculum development and integral members of the program. Using evaluation tools to collect data validated our involvement and was vital in helping us successfully advocate for more time in the classroom, thereby providing an enhanced instructional experience. Our involvement in the program demonstrated an effective method of building alliances with health professionals and community members to provide resources for nutrition education, promotion of healthy eating habits, and prevention of chronic diseases. Other librarians in health sciences could also use this approach to increase exposure to the services they provide while building healthier communities.

This collaboration has also enabled the outreach librarian to expand into other community engagement activities that have benefited the younger generation, who are themselves at risk of developing chronic health issues. While heart disease is the leading cause of death in Mobile County, community assessment data also indicate that diabetes is on the rise locally [7]. With our community confronting these health issues, having the opportunity to provide information on healthy eating habits and nutrition to college and high school students has been extremely beneficial.

Most importantly, this collaboration helped community members learn the importance of healthy eating and meal preparation. When community members have access to current and reliable information that can be easily understood, they become better informed, which can lead to more engaging conversations with their health care providers. Thus, patient education has huge potential for improving the quality of care and resulting in better health outcomes [8].

The emergence of culinary medicine programs has addressed the inadequacies of nutrition education in the curricula of medical, nursing, and health sciences schools, where content focuses more on traditional science-based disciplines such as anatomy than food-related knowledge and skills [1, 2, 4, 9, 10]. Additionally, with the rise in obesity and other chronic diseases, the impetus to improve preventive medicine among health professionals and their competency in clinical nutrition becomes imperative [2].

The Goldring Center has led the way to significant changes in the medical school curriculum and has proved to be critical in medical students' competency in providing nutrition education and nutrition counseling to their patients [2]. The culinary medicine initiative has broadened to include nursing, allied health, and other health sciences departments, which in turn have partnered with culinary schools, chefs, and dieticians to give health sciences students the knowledge to prepare and cook tasty food while learning about its nutritional value. This, in turn, has provided them with the skills to communicate with their patients the importance of healthy eating choices that prevent and treat diseases that are diet related [9]. Our opportunity to partner with the MCI Culinary Medicine program has enabled us to bring awareness of quality consumer health resources that can be used to enhance these patient provider conversations. In addition, the exposure to these resources has planted the seed for continued use by community members for their health information needs.

Health sciences librarians have always supported the research needs of health sciences students and the professional community, but in our case, these constituencies saw how easy-to-understand consumer health resources could be incorporated into their patient consultations. Culinary medicine provides students with the skills to counsel patients in preventive health behaviors that enhance a patient's self-care and well-being. With our contribution, they have learned about new tools to assist in this endeavor [1]. Our demonstration of how to locate recipes using MedlinePlus to community members brought the use of reputable library resources into the kitchen, and the medical student instructor in the first cohort saw the ease with which they could be used for counseling patients. Thus, the hands-on approach to dietary counseling and education was enriched by our involvement.

With the implementation of culinary medicine, physicians, nurses, and other health care providers can help patients make sound medical decisions about their eating habits in a way that they understand best: through the foods they eat [1, 9]. Providers can begin conversations with patients on a practical level and be more confident in the information that they provide. Patients can learn how to eat healthier and be more participatory in their well-being, and librarians can be seen as a positive force in culinary medicine education by providing essential information for all. We hope that other culinary medicine collaborations will enlist the participation of health sciences librarians to further enhance the overall effectiveness of this exciting development in health education and patient empowerment.

## Data Availability

Data associated with this article are available in Figshare at 10.6084/m9.figshare.12374264.v1 and 10.6084/m9.figshare.12374261.v1.

## SUPPLEMENTAL FILE

**Appendix:** University of South Alabama (USA) Mitchell Cancer Institute (MCI) Culinary Medicine: MedlinePlus introduction: evaluation survey
