# Supplementary material for: Health sciences librarians supporting health and nutrition education in a culinary medicine curriculum
Source: J Med Libr Assoc. 2020 Oct 1;108(4):631–8. doi: 10.5195/jmla.2020.911 (PMC7524631; doi:10.5195/jmla.2020.911)
Supplement: Supplementary file 1 — Appendix: University of South Alabama (USA) Mitchell Cancer Institute (MCI) Culinary Medicine: MedlinePlus introduction: evaluation survey [file jmla-108-4-631-s01.pdf]

JMLA Journal of the Medical Library Association

Supplemental content to
J Med Libr Assoc. Oct;108(4):dx.doi.org/10.5195/jmla.2020.911
www.jmla.mlanet.org
© Lemley, Fenske 2020

Health sciences librarians supporting health and nutrition education in a culinary medicine curriculum

Trey Lemley, MLIS, JD; Rachel Finch Fenske, MLS

APPENDIX

University of South Alabama (USA) Mitchell Cancer Institute (MCI) Culinary Medicine: MedlinePlus introduction: evaluation survey

This is a confidential questionnaire to assess and improve the training provided to you. Summaries of course feedback may be reported publicly, but your specific responses will not be reported. Your participation is voluntary, but we value your assessment and hope you will complete the survey. Thank you!

Please indicate your level of agreement to the following statements by circling your choice:

Strongly agree	Somewhat agree	Somewhat disagree	Strongly disagree	Not applicable
(1)	(2)	(3)	(4)	(5)

The session led by the librarians introduced me to one or more health information sources or tools that I had never used before.	ST Agree 1	SW Agree 2	SW Disagree 3	ST Disagree 4	Not applicable 5
I think I can apply the information I learned in the MedlinePlus introduction session to my health and nutritional needs.	ST Agree 1	SW Agree 2	SW Disagree 3	ST Disagree 4	Not applicable 5
The information presented was helpful.	ST Agree 1	SW Agree 2	SW Disagree 3	ST Disagree 4	Not applicable 5
The information presented was clear and well organized.	ST Agree 1	SW Agree 2	SW Disagree 3	ST Disagree 4	Not applicable 5
I plan to start using MedlinePlus to locate health and nutrition information.	ST Agree 1	SW Agree 2	SW Disagree 3	ST Disagree 4	Not applicable 5
I would recommend MedlinePlus to my friends and family.	ST Agree 1	SW Agree 2	SW Disagree 3	ST Disagree 4	Not applicable 5
The amount of time devoted to the MedlinePlus session was adequate.	ST Agree 1	SW Agree 2	SW Disagree 3	ST Disagree 4	Not applicable 5

Journal of the Medical Library Association
Supplemental content to
J Med Libr Assoc. Oct;108(4):dx.doi.org/10.5195/jmla.2020.911
www.jmla.mlanet.org
© Lemley, Fenske 2020

I would like to have more time learning how to use MedlinePlus.	ST Agree 1	SW Agree 2	SW Disagree 3	ST Disagree 4	Not applicable 5
I would like to have a hands-on session using MedlinePlus in the Culinary Medicine curriculum.	ST Agree 1	SW Agree 2	SW Disagree 3	ST Disagree 4	Not applicable 5
Having the library component in the MCI Culinary Medicine curriculum was beneficial.	ST Agree 1	SW Agree 2	SW Disagree 3	ST Disagree 4	Not applicable 5
